# Endophytic microbial community in two transgenic maize genotypes and in their near-isogenic non-transgenic maize genotype

**DOI:** 10.1186/s12866-014-0332-1

**Published:** 2014-12-24

**Authors:** Débora Alves Ferreira da Silva, Simone Raposo Cotta, Renata Estebanez Vollú, Diogo de Azevedo Jurelevicius, Joana Montezano Marques, Ivanildo Evódio Marriel, Lucy Seldin

**Affiliations:** Universidade Federal do Rio de Janeiro, Instituto de Microbiologia Paulo de Góes, Rio de Janeiro, RJ Brazil; Departamento de Ciências do Solo, Escola Superior de Agricultura Luiz de Queiroz, Universidade de São Paulo, Piracicaba, São Paulo Brazil; EMBRAPA/CNPMS- Empresa Brasileira de Pesquisa Agropecuária, Centro Nacional de Pesquisas de Milho e Sorgo, Sete Lagoas, MG Brazil; Laboratório de Genética Microbiana, Instituto de Microbiologia Paulo de Góes, Universidade Federal do Rio de Janeiro, Centro de Ciências da Saúde, Bloco I, Ilha do Fundão, Rio de Janeiro, RJ CEP 21941-590 Brazil

**Keywords:** Genetically modified maize, Near-isogenic non-transgenic maize, Endophytic microbial community, PAT enzyme, Cry proteins

## Abstract

**Background:**

Despite all the benefits assigned to the genetically modified plants, there are still no sufficient data available in literature concerning the possible effects on the microbial communities associated with these plants. Therefore, this study was aimed at examining the effects of the genetic modifications of two transgenic maize genotypes (MON810 – expressing the insecticidal Bt-toxin and TC1507 – expressing the insecticidal Bt-toxin and the herbicide resistance PAT [phosphinothricin-*N*-acetyltransferase]) on their endophytic microbial communities, in comparison to the microbial community found in the near-isogenic non-transgenic maize (control).

**Results:**

The structure of the endophytic communities (Bacteria, Archaea and fungi) and their composition (Bacteria) were evaluated by denaturing gradient gel electrophoresis (DGGE) and the construction of clone libraries, respectively. DGGE analysis and the clone libraries of the bacterial community showed that genotype TC1507 slightly differed from the other two genotypes. Genotype TC1507 showed a higher diversity within its endophytic bacterial community when compared to the other genotypes. Although some bacterial genera were found in all genotypes, such as the genera *Burkholderia*, *Achromobacer* and *Stenotrophomonas*, some were unique to genotype TC1507. Moreover, OTUs associated with *Enterobacter* predominated only in TC1507 clone libraries.

**Conclusion:**

The endophytic bacterial community of the maize genotype TC1507 differed from the communities of the maize genotype MON810 and of their near-isogenic parental genotypes (non-Bt or control). The differences observed among the maize genotypes studied may be associated with insertion of the gene coding for the protein PAT present only in the transgenic genotype TC1507.

## Background

Endophytic microorganisms are conventionally defined as bacteria, archaea or fungi that inhabit plant tissues for at least a period in their life cycle, and cause no negative effects on their hosts [1]. They are often described as an important modulating agent of plants’ fitness, promoting the health of the host plants and their ability to adapt to environmental stresses. The promotion of host growth may occur through nitrogen fixation, enhanced availability of nutrients (phosphorus and iron), suppressing pathogens, auxin production, among others [2-4].

The factors that drive the assembly of the endophytic community have not been completely established. Bacterial root colonization often starts with the recognition of specific compounds in the root exudates by the bacteria, and endophytic colonization tends to follow a stochastic pattern [4]. It is well known that biotic (plant genotype, taxonomic identity and specific plant traits) and abiotic (soil characteristics, temperature, seasonality, among others) characteristics can influence the assembly of the endophytic community [5,6]. Using green-fluorescent-protein (GFP)-labeled endophytes, Johnston-Monje and Raizada [7] demonstrated the transport of the endophytes from seeds into plant roots and tissues. Also, endophytes injected into stems moved into the roots and rhizosphere, suggesting that there may be a continuing microbial community shifts within the root microbiome [4]. Moreover, agricultural management practices introduced to the environment, such as herbicide and pesticide applications, crop management and genetically modified (GM) plants, can also influence plant-microbe interactions and may result on a variation of the diversity, structure and richness of the microbial community that will live inside the plant.

Worldwide, cultivation of GM plant is growing impressively. The reduced need for application of broad-spectrum chemical pesticides, such as in the case of maize carrying *Bacillus thuringiensis* (Bt) insect toxin genes, is undoubtedly of great advantage [8,9]. Brazil achieved, for the fourth consecutive year, the largest increase in GM crop area of ~6.3 million hectares compared to 2011. GM maize remained the second most important crop, with a total of 12.1 million hectares for both summer (5.2 million hectares) and winter (6.9 million hectares) [10].

While the use of GM plants increases, different studies were performed to determine whether GM plants affect microbial species. Nevertheless, this is still a controversial matter [9,11-14]. In a previous study, the bacterial and fungal communities associated with the rhizosphere of two genetically modified (GM) maize genotypes by inclusion of transgenes were compared by culture-independent methodologies to the non-Bt near-isogenic parental genotype. Two different Brazilian soils and three stages of plant development in two different periods of the year were included in the study [14]. No discernible effects of the GM genotypes, as compared to the non-Bt genotype, were observed; but clear differences occurred in these rhizosphere communities between the soils and the periods of the year in which maize was cultivated [14]. However, the effect of host genome modifications on their endophytic microbial communities has not been considered. For this reason, this study was aimed at comparing the GM genotypes with the near-isogenic maize hybrid to consider the influence of the host genome modification in their endophytic communities. We hypothesize that the introduction of exogenous genes (Cry1F and Cry1Ab encoding genes from *Bacillus thuringiensis* and the herbicide resistance phosphinothricin-*N*-acetyltransferase [*pat* gene] from *Streptomyces viridochromogenes*) in maize may alter the endophytic microbial communities associated with these plants due to a possible modification of their root exudation pattern. Molecular approaches (denaturing gradient gel electrophoresis and clone libraries) were used to determine the structure of endophytic bacterial, archaeal and fungal communities and the composition of endophytic bacterial communities in the roots of maize genotypes, respectively.

## Results

### Structure of endophytic bacterial, archaeal and fungal communities in the roots of maize genotypes analyzed by DGGE

The three maize genotypes (non-Bt, MON810 and TC1507) were sampled after 90 days of growing in Cerrado soil, during the raining season in Brazil. They did not show any apparent difference in growth conditions or productivity. No signs of disease or nutrient scarcity were noted (data not shown).

**Figure 1:**
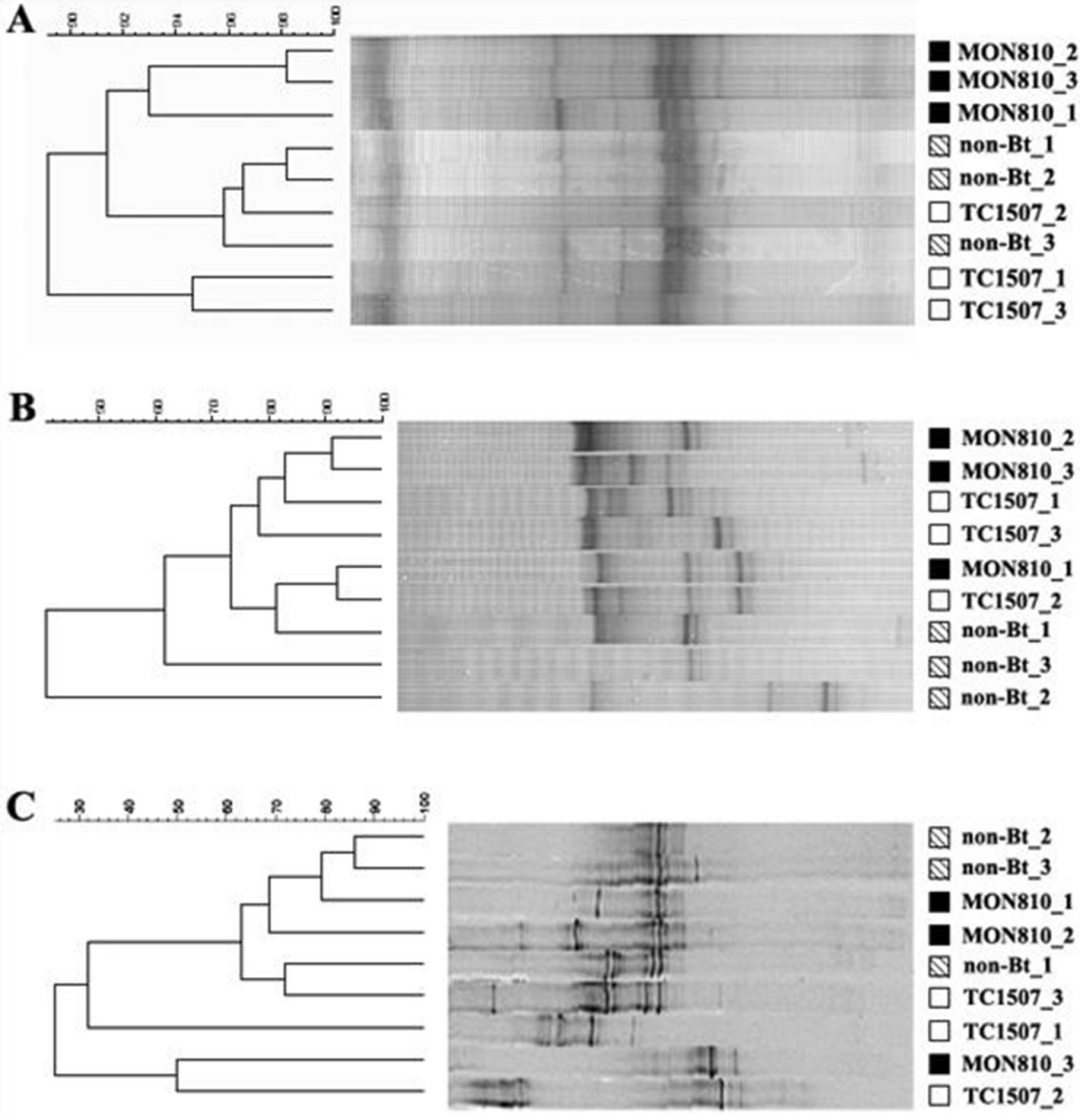
**Denaturing gradient gel electrophoresis (DGGE) fingerprints of bacterial (A), archaeal (B) 16S rRNA gene fragments, and fungal (C) internal transcribed spacer (ITS) region amplified from the DNA extracted from the three maize genotypes (non-Bt = control, MON8102 and TC1507) and their respective dendrograms constructed using the Pearson correlation index**. Cluster analysis was performed by the unweighted pair group method with average linkages (UPGMA) using the BioNumerics software.

The DGGE profiles obtained from the endophytic bacterial community were found to be very similar among the two transgenic genotypes and their near-isogenic non-transgenic maize (control) (≥89% similarity). However, samples obtained from the control and MON810 were closer between each other than those obtained from TC1507 (Figure 1A). Common bands were detected in all samples. Cluster analysis corroborated the visual interpretation of the DGGE profiles, where three main groups were formed: A (three replicates of MON810), B (three replicates of the control and one sample of TC1507) and C (two replicates of TC1507).

Endophytic archaeal community found in the two transgenic genotypes showed to be closer (separated in 74% similarity) between each other when compared to that of the control (Figure 1B). In fact, the three replicates of the control were not as reproducible as observed in the triplicate samples from other communities evaluated in our study (Figure 1B). An opposite situation could be observed when the endophytic fungal community was considered (Figure 1C). Reproducible replicates of the control were observed while those from the two transgenic genotypes were quite variable. Fungal community of MON810 and TC1507 showed between 10 to 75% similarity (Figure 1C), suggesting little influence of the genotypes as no separate clusters were observed.

### Diversity of the endophytic bacterial community in the roots of the two transgenic genotypes and their near-isogenic non-transgenic maize

The DNA extracted from the roots of the three maize genotypes (non-Bt, MON810 and TC1507) was used to construct nine 16S rRNA gene clone libraries. More than 790 high quality sequences were obtained, and these sequences (approximately 433 bp without the primer sequences) were further clustered into OTUs with 97% nucleotide similarity.

The data obtained from the statistical analyses showed that the clone libraries covered 87 to 89% of the bacterial 16S rRNA phylotypes that resulted from the PCR amplification using the three maize genotypes (Table 1). The highest richness (Chao 1) and diversity (Shannon) of 16S rRNA phylotypes were observed in the clone libraries constructed with PCR amplification products obtained from MON810 and TC1507, respectively (Table 1). However, the values obtained in both indices for the three libraries (control, MON810 and TC1507) were not statistically significant (*t* test).

**Table 1:** **Coverage, Shannon-Weaver and Chao1 diversity indices based on the bacterial 16S rRNA-coding sequences in the clone libraries obtained from the three maize genotypes**

**Clone libraries**	**Number of clones**	**Number of OTUs**	**Chao1**	**Shannon-Weaver**	**Coverage (%)**
Control	267	32	43.7 ± 12.5	1.64 ± 0.17	0.87 ± 0.02
MON810	255	26	46 ± 29.6	1.6 ± 0.28	0.88 ± 0.05
TC1507	274	28	31.7 ± 3.3	2.18 ± 0.14	0.89 ± 0

± − standard deviation of the three values obtained (three clone libraries per genotype).

**Figure 2:**
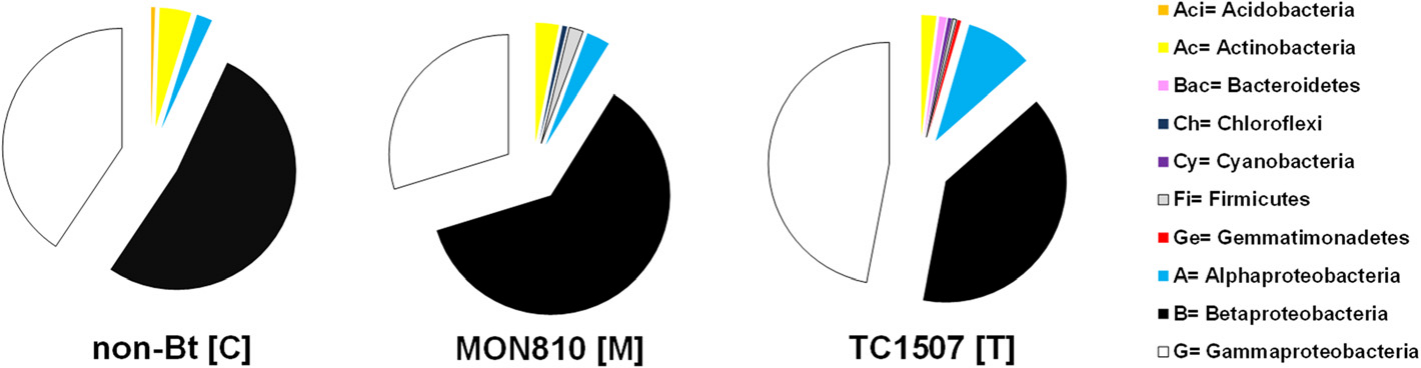
**Relative abundance of bacterial phyla and of Proteobacteria classes in the clone libraries.** Each graph represents the sum of three libraries per maize genotype.

Phylogenetic analyses of bacterial 16S rRNA-coding genes revealed a strong relative abundance of OTUs associated with the phylum Proteobacteria (control – 95.2%, MON810 – 94.3% and TC1507 – 95.5%). However, the relative abundance of Proteobacteria classes was different among maize genotypes. Gammaproteobacteria represented the main bacterial class of TC1507 genotype (46.7%), whereas Betaproteobacteria were more abundant in non-Bt and MON810 genotypes (51.8 and 61.4%, respectively) (Figure 2). Alphaproteobacteria represented 1% of OTUs obtained from non-Bt and MON810 genotypes, and 6% of OTUs in TC1507. Furthermore, OTUs associated with the phylum Actinobacteria were also observed in all genotypes, but corresponded to only 2.1 to 4.5% of the OTUs (Figure 2). Some other phyla were also found in at least one of the clone libraries obtained from the three maize genotypes, such as Gemmatimonadetes, Bacteroidetes, Acidobacteria and Chloroflexi (Figure 2). A systematical analysis of taxonomic groups (from phylum to genus) among all genotypes was further carried out. Table 2 gives an overview of the abundance of different bacterial groups in the clone libraries obtained. OTUs associated with *Stenotrophomonas*, *Achromobacter* and *Burkholderia* genera were found in all clone libraries. OTUs associated with *Enterobacter* and *Rhizobium* were dominant in TC1507 clone libraries, but not in those of MON810 and non-Bt (Table 2). In addition, OTUs associated with other genera were observed within each genotype; however, their relative abundances were always lower than 2%.

**Table 2:** **Relative abundance of bacterial genera among transgenic and non-transgenic maize**

		**Relative abundance of bacterial genera**		
		**Samples**		
Bacterialphyla (or Proteobacteria classes)	Genera	**non-Bt [C]**	**MON810 [M]**	**TC1507 [T]**
Acidobacteria	Unclassified Holophagae	0.54%	-	-
Actinobacteria	Unclassified Acidimicrobineae	0.54%	-	-
	Unclassified Frankineae	-	0.63%	-
	Unclassified Micrococcineae	0.54%	1.27%	1.00%
	Unclassified Micromonosporineae	1.07%	-	-
	Unclassified Propionibacterineae	-	0.63%	0.50%
	Unclassified Pseudonocardineae	0.54%	-	-
	Unclassified Streptomycineae	-	-	0.50%
	Unclassified Streptosporangineae	0.54%	-	-
	Unclassified Rubrobacteridae	0.54%	-	-
	Unclassified Conexibacteraceae	0.54%	0.63%	-
Bacteroidetes	*Chryseobacterium*	-	-	0.50%
	*Flavobacterium*	-	-	0.50%
Chloroflexi	unclassified SHA-26	-	0.63%	-
Cyanobacteria	Chloroplast	-	-	0.50%
Firmicutes	*Paenibacillus*	-	1.90%	-
	*Lactococcus*	-	-	0.50%
Gemmatimonadetes	unclassified S0134	-	-	0.50%
Alphaproteobacteria	*Ochrobactrum*	-	-	0.50%
	*Ancylobacter*	0.54%	-	-
	*Pedomicrobium*	0.54%	-	-
	*Phyllobacterium*	-^a^	2.54%^b^	0.50%^a^
	*Rhizobium*	-^a^	-^a^	7.51%^b^
	*Parvibaculum*	0.54%	-	-
	Shinella_genera_incertae_sedis	-	-	0.50%
	*Paracraurococcus*	-	0.63%	-
	unclassified MND8	0.54%	-	-
Betaproteobacteria	*Achromobacter*	32.64%^a^	36.12%^a^	13.51%^b^
	*Bordetella*	1.61%	1.27%	1.00%
	GKS98	1.61%	1.90%	0.50%
	*Pusillimonas*	0.54%	-	-
	Uncultured Alcaligenaceae	2.68%	1.27%	1.00%
	*Burkholderia*	11.24%	15.84%	18.52%
	*Limnobacter*	-	-	1.00%
	*Caenimonas*	-	-	0.50%
	*Comamonas*	-	-	0.50%
	*Inhella*	-	1.27%	-
	*Duganella*	-	0.63%	-
	*Herbaspirillum*	-	-	2.00%
	*Massillia*	-	-	0.50%
	*Naxibacter*	0.54%	2.53%	0.50%
	*Silvimonas*	1.61%	0.63%	-
Gammaproteobacteria	*Enterobacter*	1.07%^a^	1.90%^a^	23.02%^b^
	*Acinetobacter*	0.54%	-	2.50%
	*Pseudomonas*	-	-	1.50%
	*Aspromonas*	0.54%	-	-
	*Lysobacter*	0.54%	-	-
	*Rhodanobacter*	0.54%	-	-
	*Stenotrophomonas*	37.45%^a^	27.88%^ab^	20.02%^b^

-, not detected.

Different letters in superscript, following values in percentage, indicate statistical significance differences.

Mantel test analysis was performed to compare the relative abundance of the dominant bacterial community found in the different maize genotypes. Although sequences associated with *Burkholderia* were found in all libraries, their relative abundances were not statistically different among the three genotypes. On the other hand, the relative abundance of *Achromobacter*-related OTUs did not differ between non-Bt and MON810 genotypes, but it was lower in TC1507 clone libraries (p < 0.05). The relative abundance of *Stenotrophomonas*-related OTUs was statistically different only between non-Bt and TC1507 genotypes. Additionally, the relative abundances of both *Enterobacter*- and *Rhizobium*-related OTUs were higher (p < 0.05) in TC1507 clone libraries, when compared to those of MON810 and non-Bt (Table 2).

**Figure 3:**
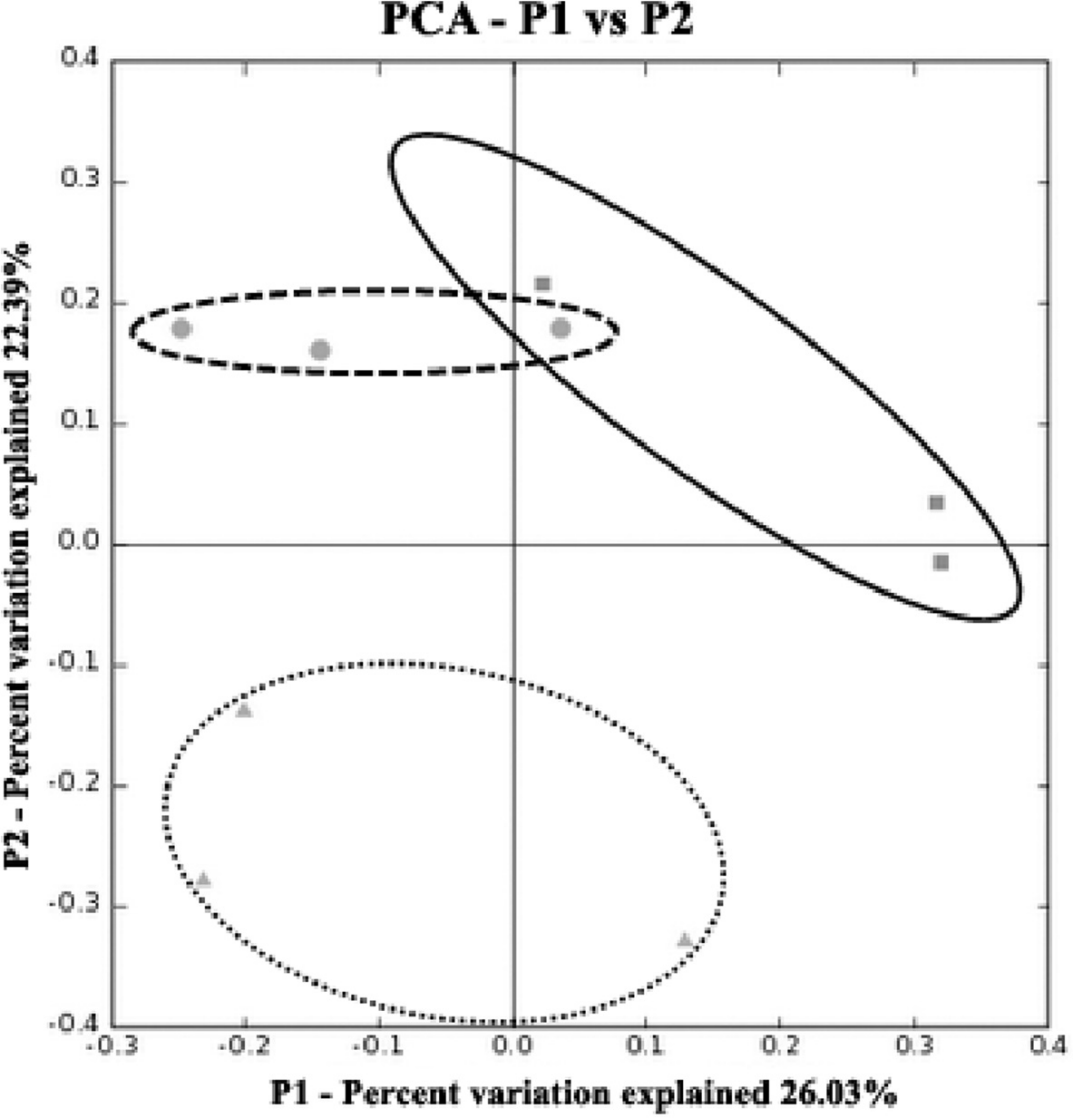
**Principal Component analyses (PCA) of the clone libraries.** The symbols correspond to the maize genotypes: ▲ TC1507; ● MON810 and ■ control (non-Bt).

The Principal Component Analysis (PCA) based on weighted UniFrac distance was also performed to analyze bacterial community structure from the three maize genotypes (Figure 3). Similar trends regarding UPGMA cluster analysis (based on Pearson correlation) were revealed. The clone libraries obtained from TC1507 tended to separate from those of the control and MON810. Conversely, the clone libraries derived from the control and MON810 could neither be separated by first-axis nor second-axis (Figure 3). These results suggest that the endophytic bacterial community of the maize genotype TC1507 (which expresses the insecticidal Bt-toxin Cry1F and the herbicide resistance phosphinothricin-*N*-acetyltransferase) differs from the communities of the maize genotype MON810 (which expresses the insecticidal Bt-toxin Cry1Ab) and the communities of the near-isogenic parental genotypes (non-Bt or control) of both GM maize samples.

## Discussion

In a previous study, Cotta et al. [14] evaluated the possible effects of the same two Bt maize plants used here, but cultivated in two different tropical soil types during two different periods of cultivation and during different plant growth stages, in relation to the diversities of their rhizospheric microbial communities. No major effects of the Cry-gene-loaded GM maize plants on the bacterial, actinobacterial and fungal communities were observed. However, no data were presented concerning their endophytic microbial communities.

Saxena and Stotzky [15] and Devare et al. [16] have previously presented a finding similar to those obtained by Cotta et al. [14] when different microbial communities of the rhizosphere of transgenic and non-transgenic maize were compared. Oppositely, Stuart et al. [12] found that the fungal community was affected in transgenic imidazolinone-tolerant sugarcane plants. However, these effects were also related to other factors such as plant age. In fact, different studies have already demonstrated that factors like soil type, plant age, period of cultivation and field heterogeneity may interfere more in the structure and/or composition of the root associated-microbial community than the insertion of an exogenous gene in the plant ([17-19]; and others).

In this study, two maize genotypes (MON810, expressing the Bt-toxin Cry1Ab, and TC1507, expressing the Bt-toxin Cry1F and protein PAT) and their near-isogenic parental genotype (non-Bt) were used. They were sown in the same tropical soil and field, and they had the same developmental stage during the sampling of plants. Therefore, the influence of the major biotic and abiotic factors could be disregarded. Although many studies have already revealed that the plant genotype is an important determinant of endophyte community formation [18,20,21], the effect of host genome modification on their endophytic communities was considered here, since no previous study was available using these maize genotypes, as previously stated. Only the genetically modified Bt maize lines containing other Bt-toxins (Cry3Bb1, Cry1A105 and Cry1Ab2) have been studied before, and no influence on the structure and functioning of root-associated endophyte communities was demonstrated [22].

DGGE profiles obtained in this study showed that the endophytic archaeal and fungal communities of the two transgenic genotypes and their near-isogenic non-transgenic maize do not seem to follow any tendency of grouping, as different patterns were erratically distributed across replicates. Therefore, we can speculate that the composition of fungal and archaeal community was not specifically influenced by the plant genotypes. On the other hand, the bacterial DGGE profiles showed a minor trend of grouping, suggesting that the protein exudation resulted from the transgenic events and/or secondary (or unknown) changes in the genetic composition of maize plants influenced the endophytic bacterial community structure. To better characterize the composition of the endophytic bacterial community within each maize genotype, different clone libraries were constructed. The relative abundance of the main bacterial genera was significantly different between the maize genotypes, reflecting a specific relationship between endophytic community and host genome. Likewise, Aira et al. [23], Gaiero et al. [4] and Donn et al. [24] demonstrated that plants of different species or even plant genotypes grown in the same soil harbor distinct groups of endophytes, suggesting that plants impact the microbial communities found inside their roots.

Prischl et al. [22] showed that members of Alphaproteobacteria, Actinobacteria and Gammaproteobacteria were the predominant bacterial community cultivated from transgenic maize expressing *Bacillus thuringiensis* Cry proteins. Within genera level, McInroy and Kloepper [25] showed that the most frequently isolated members of the endophytic bacterial community in maize were *Enterobacter* spp., followed by *Burkholderia* spp. We found that Betaproteobacteria were more abundant in non-Bt and MON810 (expressing only insecticidal Bt-toxin Cry1F). On the other hand, Gammaproteobacteria and Alphaproteobacteria were more abundant in TC1507 (expressing both Bt-toxin Cry1F and phosphinothricin-*N*-acetyltransferase). Furthermore, the relative abundance of *Enterobacter* and *Rhizobium*, from Gammaproteobacteria and Alphaproteobacteria classes, respectively, was much higher in TC1507 when compared to other genotypes. Pereira et al. [26] also observed that Gammaproteobacteria, within *Enterobacter*, *Erwinia*, *Klebsiella*, *Pseudomonas*, and *Stenotrophomonas* genera were the predominant groups in association with maize roots.

Different endophytic *Enterobacter* strains have been described as plant growth promoters [27] and it is plausible to consider that specific bacteria are attracted by the GM maize genotype TC1507. The same situation can be observed when the relative abundance of *Rhizobium* is considered. In fact, the only difference between TC1507 and MON810 genotypes is the expression of the herbicide resistance phosphinothricin-*N*-acetyltransferase [*pat* gene]. However, when we compared the bacterial community from the three maize genotypes, the results showed that those of TC1507 tended to separate from those of the control and MON810. Therefore, either the presence of the protein PAT or other minor genetic differences between the control and TC1507 resulted from the process of obtaining TC1507 genotype may be responsible for the selection of specific bacterial communities inside the roots of the transgenic maize – TC1507. Nevertheless, it is still necessary to correlate the bacterial selection observed in the genotype TC1507 at the functional level.

## Conclusion

The endophytic bacterial community of the maize genotype TC1507 differed from the communities of the maize genotype MON810 and of their near-isogenic parental genotypes (non-Bt or control). Other studies are still necessary to determine whether either secondary differences occurring upon genetic modifications or the presence of the protein PAT in the transgenic maize TC1507 is the responsible for the selection of specific bacterial communities inside its roots. Therefore, the precise impact of transgene expression on endophytic community shifts is something that needs to be further investigated.

## Methods

### Maize cultivars and experimental conditions

This study was conducted in the experimental field located at EMBRAPA Milho e Sorgo, in the city of Sete Lagoas (geographical coordinates: 19° 28’ South, 44° 15’ West), State of Minas Gerais, Brazil. The soil of this area is characterized as dark-red acid distrophic latosol, with a clayey texture (pH 5.9; silte 11 dag kg^−1^, clay 59 dag kg^−1^, sand 30 dag kg^−1^). The experimental area (6.4 m^2^) consisted of completely randomized fourfold replicated 5×5 m plots containing four rows of 5 m length, with spaces of 0.8 m between rows and 0.2 m between plants. More details about the experimental conditions are presented in Cotta et al. [14]. The maize genotypes were planted randomly in each plot, and only one seed lot was used for each maize genotype. Two hybrid Bt-maize genotypes were used: i) the Guardian hybrid (Monsanto), a GM maize cultivar which represents transformation event MON810 genotype 30F35Y (expressing about 4.5 μg g^−1^ of soil of the insecticidal Bt-toxin Cry1Ab from *Bacillus thuringiensis*), and ii) the Herculex hybrid (Pioneer), which represents the transformation event TC1507 genotype 30F35H (expressing about 5.0 μg g^−1^ of soil of the insecticidal Bt-toxin Cry1F from *Bacillus thuringiensis* and the herbicide resistance phosphinothricin-*N*-acetyltransferase [*pat* gene] from *Streptomyces viridochromogenes*). The near-isogenic parental line of both GM maize cultivars was used as the control. Sampling was performed during grain filling and maturity (90 days). Three plants of each cultivar were harvested and their roots were sterilized, as described by Monteiro et al. [28]. Briefly, root samples were washed in distilled water to remove the attached soil, and were surface sterilized by UV light exposure for 30 min, washed with 70% ethanol for 5 min, 4-6% sodium hypochloride for 5 min, 70% ethanol for 2 min, and rinsed four times with sterile water [28]. To check the efficiency of the disinfection procedure, 100 μl of the water used in the last washing was plated onto Trypticase Soy Broth (TSB)-containing plates with 0.1% nistatine (100 μg ml^−1^), and incubated for 5 days at 32°C. Roots that were not contaminated, as detected by the culture-dependent sterility test, were used for further DNA isolation.

### DNA extraction of endophytic community

Roots were cut into pieces of approximately 0.3 cm, homogenized in a mortar with a pestle, and then 500 mg of each triplicate/maize genotype was submitted to total microbial community DNA extraction using the FastPrep Spin kit for soil DNA (MP Biomedicals, Solon, OH, USA). DNA preparations were visualized by electrophoresis in a 0.8% agarose gel in 1x TBE buffer [29] to assess yield and integrity. Samples were then stored at −20°C prior to PCR amplifications.

### PCR-DGGE analysis of bacterial, archaeal and fungal communities

A nested PCR approach was used to amplify the bacterial and archaeal 16S rRNA gene sequences and the fungal internal transcribed spacer (ITS) region from surface sterilized roots. Briefly, for the PCR amplification of the genes encoding bacterial 16S rRNA, the primers F799 and R1492 [30] were used for the first round of PCR, and the primers U968F-GC and L1401R [31] were later used for the second-round amplification. To amplify the archaeal 16S rRNA gene sequences, the primers Arch21f and Arch958r [32] were used for the first round of PCR. The primers Arch344f-GC and Arch519r ([33] and [34], respectively) were then used for the second-round amplification. For the amplification of the fungal ITS region, the first PCR reaction was performed using the primers EF4F and ITS4R ([35] and [36], respectively]). Amplicons obtained in this first PCR reaction were then used as templates for a second amplification procedure using the primers ITS1F-GC and ITS2R ([37] and [36], respectively). The PCR conditions were those previously described in the references cited above. In all cases, 1 μl of template DNA (50 to 100 ng) was used in the first-round of PCR, whereas a 1:100 dilution of the first-round of PCR product was used as a template for the second-round amplification.

DGGEs were performed using an INGENYphorU-2system (Ingeny International BV, Goes, The Netherlands). PCR products (approximately 300 ng) were applied directly to 8% (w/v) polyacrylamide gels in 1X TAE buffer (40 mM Tris-acetate [pH 8.3] and 1 mM disodium EDTA) containing a denaturing gradient of urea and formamide varying from either 46.5 to 60% (total Bacteria and Archaea) or 30 to 70% (fungal community). The gels were run for 17 h at 60°C and 140 V. After electrophoresis, the gels were silver-stained according to Heuer et al. [38]. The dendrograms were constructed after image capture and analysis using the Pearson correlation index for each pair of lanes within a gel. Cluster analysis was performed by the unweighted pair group method with average linkages (UPGMA) using the BioNumerics software.

### Construction of clone libraries and phylogenetic analysis

Fragments of PCR-amplified bacterial 16S rRNA gene (using the protocol described above without GC clamp) were obtained from each maize genotype. Amplicons were purified using the Wizard^™^system (Promega), and cloned using the InsTAclone^™^ (Fermentas, Sinapse Biotecnologia, SP, Brazil), following instructions of supplier. After the transformation of *Escherichia coli* JM109 competent cells, clones were picked and the presence of inserts of the correct size was verified by PCR using the primers M13F and M13R. The sequencing of the selected clones was performed using Macrogen (South Korea) facilities. The sequences were compared using BlastN facility (NCBI), aligned using ClustalX, and edited using Bioedit software (Ibis Biosciences Inc., CA, USA). The alignments were deposited in TreeBase (ID: 16762). The clone libraries were compared using MOTHUR software [39], and classified into operational taxonomic units (OTUs) with 97% similarity. These sequences were deposited in the DNA Data Bank of Japan [DDBJ: AB971452 - AB971537]. The OTU-generated matrices were used to calculate the richness of the species using Chao1 estimators [40] and the Shannon-Weaver diversity index [41]. Coverage (C) was also calculated, where C equals 1 − n1/N, and n1/N is the ratio of clones that appeared only once (n1) to the total number of clones (N) [30]. The Principal Component Analysis (PCA) was done using Unifrac (http://www.unifrac.colorado.edu).
